# Perforated Giant Meckel's Diverticulum Mimicking Colonic Ischemia

**DOI:** 10.7759/cureus.3753

**Published:** 2018-12-19

**Authors:** Abdul Ahad Rana, Markus Trochsler, Harsh Kanhere

**Affiliations:** 1 Surgery, Royal Adelaide Hospital, Adelaide, AUS; 2 Surgery, The Queen Elizabeth Hospital, Adelaide, AUS

**Keywords:** giant meckel's diverticulum, pseudoischemia, meckel's diverticulitis, perforation

## Abstract

Meckel's Diverticulum is one of the most common congenital anomalies of the gastrointestinal tract. However, its presentation as a complicated Giant Meckel's Diverticulum in an adult is rare. We present a case of a perforated Giant Meckel’s mimicking ischemia of the right colon. This case report highlights the importance of having a high index of suspicion for this rare diagnosis.

## Introduction

Meckel's Diverticulum (MD) is one of the most common congenital anomalies of the gastrointestinal tract. It is formed as a result of an incomplete obliteration of the vitelline duct in about 2% of the population [1, 2]. MD is a true diverticulum and is usually located within 60 to 100 cm proximal to the ileocaecal valve [3]. Cells lining the MD are pluripotent, and can differentiate into gastric, pancreatic or colonic mucosa. MD is reported to measure between 2 and 3 cm in length. Diverticulitis of more than 5 cm in length are defined as Giant Meckel's Diverticulum [4]. Giant MD is rare with very few case reports published in the literature [1-6].

Patients with giant MD usually remain asymptomatic; however, they have a four to six percent lifetime risk of developing complications [3]. Males are more prone to develop complications than females and are therefore more likely to be diagnosed [5]. Complications include inflammation, perforation, intussusception, volvulus and hemorrhage [3].

We present a case of a perforated giant MD mimicking the clinical presentation of colonic perforation secondary to ischemia of the ascending colon.

## Case presentation

We present a case of a 50-year-old Caucasian male who presented to the emergency department with complaints of lower abdominal pain, fever and sweating. On examination, the patient had tenderness to palpation in the right iliac fossa, with significant rebound tenderness and guarding. Body temperature was recorded at 38.5°C.

The patient's past medical history was significant for an incident of similar pain six months prior to presentation. He was diagnosed with sigmoid diverticular disease confirmed by computed tomography (CT) scan and managed conservatively. A subsequent colonoscopy confirmed the diagnosis, and did not reveal any other colonic pathology.

**Figure 1:**
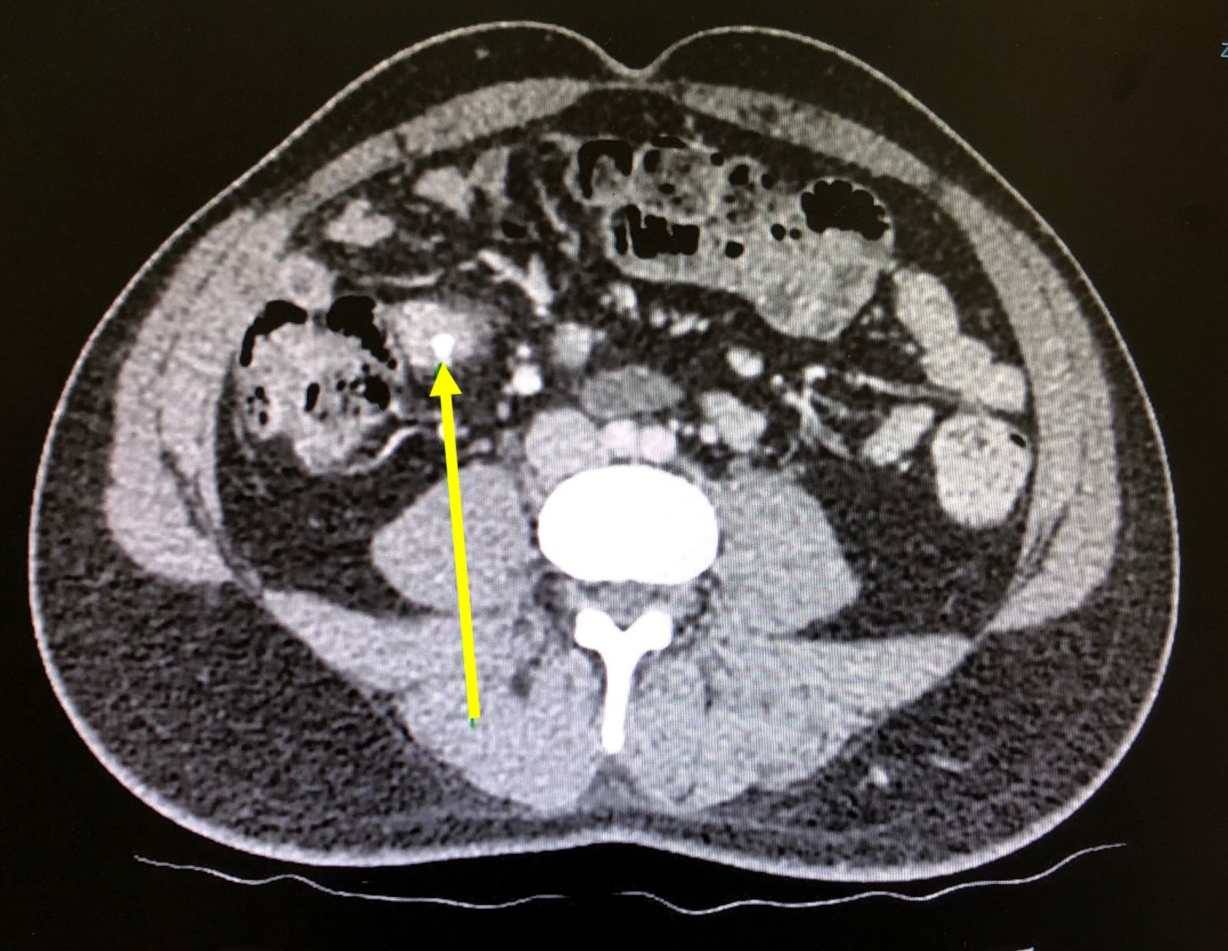
Blind ending structure with calcification (arrow) and surrounding fat stranding that appears to be arising from a loop of distal small bowel, representing Meckel’s diverticulitis.

Further investigations revealed a raised white blood cell count of 16,000 per microliter. CT scan of the abdomen and pelvis disclosed evidence of extensive free gas under the right dome of the diaphragm confirming suspicion of a perforation. Fluid-filled prominent loops of small bowel were noted. However, none of them were dilated to suggest obstruction. Mild bowel thickening was also noted around the cecum. Pneumatosis coli suggestive of ischemic bowel, extending from the cecum to the proximal ascending colon was seen. A blind ending structure with calcifications was also seen (Figure 1). Other significant findings on the scan included a liver cyst, consistent with the patient’s previous CT scan, and consolidation at the base of the right lung.

**Figure 2:**
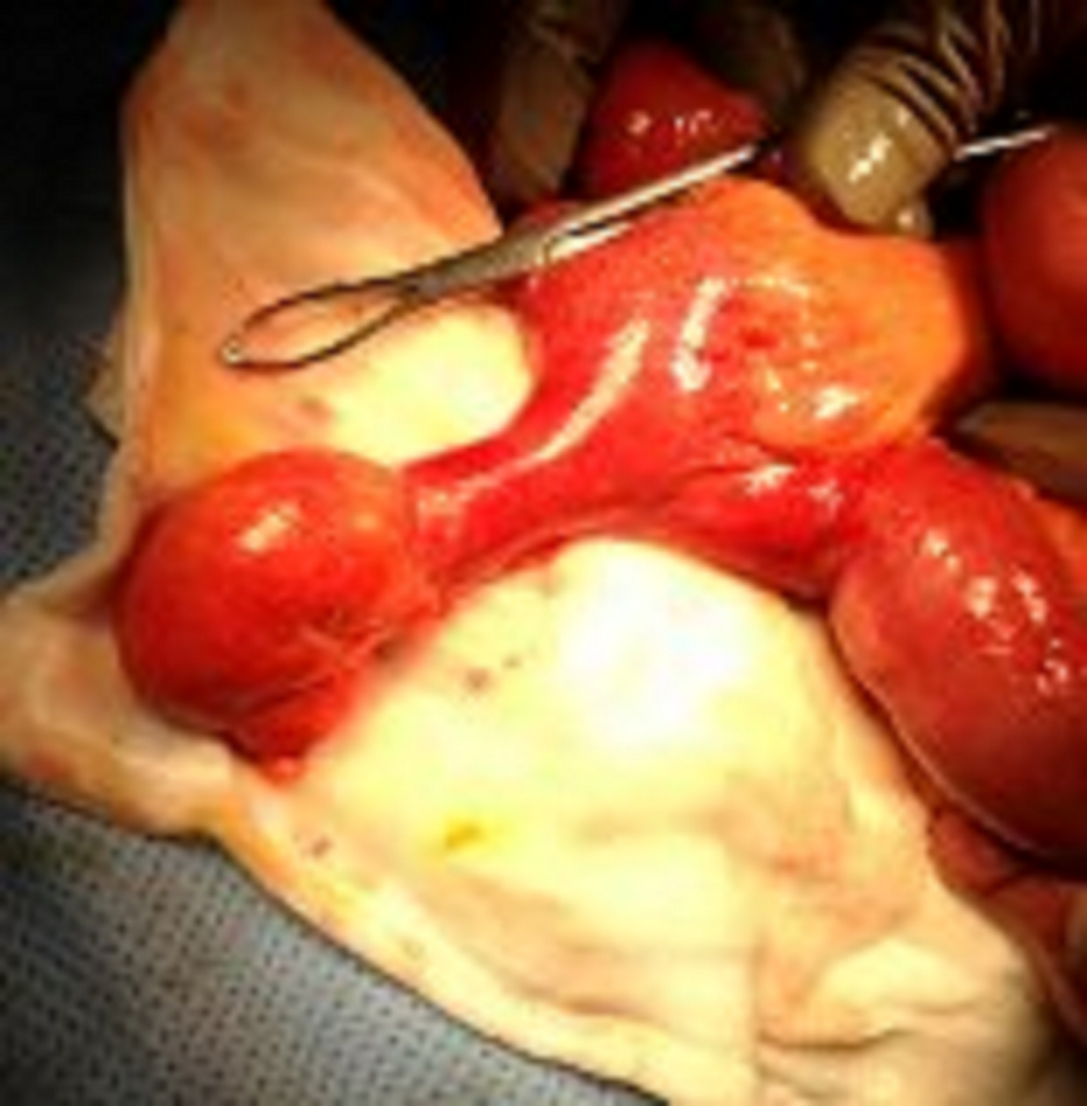
Giant Meckel's diverticulum measuring approximately 10 cm in length and 2.5 cm in width.

Subsequently, the patient underwent an emergency laparotomy and a diffuse four-quadrant peritonitis was seen. A giant perforated MD, 80 cm proximal of the ileocecal valve was identified as the cause of the peritonitis. The giant MD measured approximately 10 cm in length and 2.5 cm in width (Figure 2). A small perforation at the tip of the MD was observed. No other intraabdominal pathology was identified, in particular, there was no evidence of colonic ishchaemia. Resection of the segment of small bowel bearing the MD was performed with a side-to-side stapled anastomosis.

Pathologic examination showed a T-shaped length of bowel, 6 x 5 x 3 cm in maximal dimension, with a 3 x 5 x 6 cm portion of mesentery attached. The presumed diverticulum was 5 cm long with a maximal inner circumference of 5 cm. The perforation measured 0.2 cm and the lumen of the specimen was stained green. No heterotropic tissue was identified.

Post-operative recovery was uneventful and the patient was discharged on oral antibiotics.

## Discussion

A high index of suspicion is required for the diagnosis of Meckel's Diverticulum as a delay in the diagnosis of a complicated Meckel's Diverticulum can result in significant morbidity and mortality [1]. In our case, the initial pre-operative diagnosis was an ischemic bowel; however, during surgery, a Giant Meckel's Diverticulum was discovered. Emphasizing the importance of looking out for an MD complication in adult population is imperative in order to guarantee a safe surgical outcome. Also, this can help in planning a laparoscopic procedure instead of doing an open laparotomy.

The advantages of a laparoscopic procedure have been compared to open procedures in numerous studies and have shown relatively low early and late post-operative complications and early recovery. Centers which have specialized laparoscopic resection capabilities can easily perform resections through this method. Techniques as specialized as intra-abdominal wedge resection and extracorporeal or intracorporeal bowel segment resection have been reported in centers [1].

The diagnosis of a complicated MD is difficult, and only approximately 10% of cases are diagnosed before surgery [3]. Radiological images, ultrasonography and computed tomography can greatly assist in pre-operative diagnosis of a symptomatic MD. Scintigraphic (99Tm-pertechnetate) localization of gastric mucosa can also aid in the diagnosis [3].

The symptoms of a complicated MD start as a generalized abdominal pain which then shifts and relocates itself to the right iliac fossa. A study reported that the delay in time it takes for the pain to relocate can also raise the suspicion of a complicated MD higher up in the differential [6]. Male patients with an MD are more prone to its complications than female patients, and thus are more likely to be diagnosed [5]. In our center, we are also reporting a case of a male patient.

In addition, complicated Meckel's Diverticulitis is mostly a disease of the western world and the incidence appears to be increasing [7]. It is important to note that this case report along with those previously reported can help develop a strategy to make an early diagnosis.

## Conclusions

The ever famous "rule of two's" of MD is now in jeopardy. Our case report along with several reference articles written on giant Meckel's Diverticulitis together provides a sound ground for physicians to have a high index of suspicion of the diagnosis of a complicated MD. This will not only help them strategize pre-operatively but also have a remarkable effect in improving the morbidity and mortality of their patients.
